# Prognosis in Nervous Diseases

**Published:** 1901-11-16

**Authors:** 


### PROGNOSIS IN NERVOUS DISEASES.

In delivering the Bradshaw Lecture before the Royal College of Physicians, Dr. Judson S. Bury discussed the subject of Prognosis in Relation to Disease of the Nervous System. He showed that an approximate forecast as to the result of a disease often presents great difficulties, which are even more prominent in relation to diseases of the nervous system than to those affecting other parts of the body. When a part of the nervous system, say a nerve cell, is attacked by a poison, the effect will depend partly upon the nature, virulence, and duration of action of the poison, and partly upon the resistance offered by the cell, and both these factors present infinite variations. Difficulties in prognosis are serious enough when we think of the matter in relation to the attacking agent or to morbid anatomy; but when we consider prognosis in relation to symptoms, the difficulties of the problem assume their most acute form, in that then they are complicated with the uncertainty introduced by the way in which these symptoms manifest themselves in different individuals. For example, with the same amount of irritation of the sensory fibres the perception of pain in one person is extreme, while in another it may be slight. Again, it has to be remembered that in nervous diseases symptoms are due as much to normal physiological activity imperfectly applied as to the actual loss of function occasioned by the lesion. Thus the foot deformities met with in infantile paralysis are partly due to the unopposed action of the healthy muscles, while the exaggerated movements of a tabetic patient depend to some extent on lowered tonus of the muscles through a cutting off of sensory impulses from the motor neurons. Furthermore some symptoms may represent in one case the active and in another case the passive or arrested phases of the disease, so that the symptoms which are of real importance in regard to prognosis may not be those which indicate the locality or extent of the injury to the neurons so much as those which testify to the phase of disease present—active or passive—and these symptoms may have a natural history of their own which calls for independent investigation, and which is not as yet adequately explained by morbid anatomy. Indeed a group of symptoms may be the only part of a disease which is known to us. Thus to one group of symptoms we give the name tetany, and to another paralysis agitans, but we know neither the site nor the nature of either of these lesions. In such cases as these prognosis depends on a thorough acquaintance with types and with aberrant forms. Much the same applies to the slight variations which may be detected even when the case is to all appearance one associated with organic change. Do we understand the nature of the temporary improvements which sometimes occur in subjects of organic disease who visit such places as Lourdes or Holywell? Such improvement is not more wonderful than the temporary improvement in insanity which follows the application of a blister to the neck. The more closely we study symptomatology the more we are struck by its complexity and prognosis depends less on our knowledge of pathology than on the accuracy of our experience with regard to symptomatology. Dr. Judson Bury protests against the too common employment of the term functional, holding that we have no right to say that cases which recover have been functional only, and of a different nature to other apparently similar cases which progress to organic disease. "Surely," he says, "it is reasonable to believe that the minute initial changes of a serious disease may be removed, and that no future developments may take place." Similarly, in regard to the term "hysterical," he thinks that increasing knowledge will tend to reduce its limits still further. Prolonged vascular spasm or some other lesion must, he says, underlie a profound anæsthesia or a contraction of the visual fields, and he asks what is meant by calling these phenomena hysterical? Dr. Judson Bury then proceeds to relate some remarkable cases of recovery from symptoms which indicated serious disease, and others in which such intermissions and even temporary improvements took place in the course of fatal diseases as to show that the march of the symptoms by no means accorded with that of the progressive lesion. It may perhaps be said that so far as regards the subject matter of his lectures, Dr. Judson Bury has not pushed matters much further, and has not helped us much to say in which cases we may make a favourable, and in which an unfavourable prognosis. But his insistance on the fact that, for all our cleverness in diagnosis, some cases which ought to have gone to the bad did in the end get well, and that in some cases which did, in fact, end badly, the varying progress of the symptoms had little obvious relation with the presumable course of the pathological changes, all teaches us extreme caution in giving an absolutely hopeless prognosis in diseases of the nervous system. The symptoms which are manifest, and by which alone we can judge of the gravity of the disease, are apparently not always the direct product nor the direct measure of the morbid changes which are going on.

